# Expression, purification and biological characterization of the extracellular domain of CD40 from *Pichia pastoris*

**DOI:** 10.1186/s12896-016-0237-1

**Published:** 2016-01-25

**Authors:** Yu Zhan, Yilei Wei, Pengfei Chen, Haohao Zhang, Dandan Liu, Jie Zhang, Rongzeng Liu, Ran Chen, Jun Zhang, Wei Mo, Xiaoren Zhang

**Affiliations:** Key Laboratory of Stem Cell Biology, Institute of Health Sciences, Shanghai Jiao Tong University School of Medicine (SJTUSM) and Shanghai Institutes for Biological Sciences (SIBS), Chinese Academy of Sciences (CAS), Rm. 1126, Biological Research Life Building A, Yueyang Rd 320, Shanghai, 200031 China; Collaborative Innovation Center of System Biomedicine, Shanghai Jiao Tong University School of Medicine, Shanghai, 200240 China; Key Laboratory of Metabolism and Molecular Medicine, Ministry of Education, Fudan University, Shanghai, China; Department of Blood Transfusion, The First Affiliated Hospital of Bengbu Medical College, Bengbu, China

**Keywords:** CD40, Protein expression and purification, *Pichia pastoris*, Autoimmune diseases, Colitis

## Abstract

**Background:**

CD40, also called Bp50, is a novel member of the TNF receptor superfamily. Based on its important role in multiple physiological and pathological processes, the CD40 signaling pathway has become a vital target for treating transplantation, autoimmune diseases and cancers. This study generated a protein fragment that disrupts this signaling pathway.

**Results:**

A DNA fragment encoding the extracellular domain of CD40 (CD40-N) has been codon-optimized and cloned into pPIC9K to create a *Pichia pastoris* expression and secretion strain. SDS-PAGE and Western blotting assays using the culture media from methanol-induced expression strains showed that recombinant CD40-N, a 27 kDa glycosylated protein, was secreted into the culture broth. The recombinant protein was purified to more than 90 % using Sephadex G-50 size-exclusion chromatography and Q Sepharose Fast Flow ion exchange. Finally, 120 mg of the protein was obtained at a relatively high purity from 3 l supernatant. Binding assay (ITC_200_ assay) shown the direct interaction of CD40-N and CD40 agonist antibody (G28-5). The bioactivity of recombinant CD40-N was confirmed by its ability to disrupt non-canonical NF-κB signaling activated by CD40 agonist antibody or CD40 ligand and to inhibit ant-CD40 agonist antibody-induced TNF-alpha expression in BJAB cells in vitro. In addition, our data indicate that the protein has curative potential in treating dextran sulfate sodium (DSS)-induced colitis in vivo.

**Conclusions:**

The results show that the experimental procedure we have developed using *P. pastoris* can be used to produce large amounts of active CD40-N for research and industrial purposes. The protein fragment we have acquired has potential to be used in research or even treating inflammation diseases such as colitis.

## Background

CD40 is a 50-kDa transmembrane protein that belongs to the TNF receptor family. It is not only expressed on antigen-presenting cells such as B cells, dendritic cells and macrophages, but is also found on endothelial cells, mast cells, fibroblast cells, tumor cells and smooth muscle cells, suggesting that it has extensive functions in different physiological contexts [1–4].

There are many downstream signaling pathways coupled to the CD40 intracellular region, such as Jak3-stat3, traf6-Erk, p38, JNK, and NF-κB signaling pathway [5]. Upon binding to CD40 ligand CD40 leads to NF-κB2 p100 processing into p52 and activates non-canonical NF-κB signaling, this is likely to be important for the transcriptional regulation of CD40 target genes in adaptive immune responses [6, 7].

During the activation of immune responses, both TCR-MHC antigen signaling and co-stimulatory signals such as B7-CD28 are required for antigen-presenting cells to activate T cells. Activated T cells express high level of CD40 ligand that interacts with CD40 on antigen-presenting cells. In turn CD40-activated non-canonical NF-κB signaling up-regulates B7 expression on antigen-presenting cells, thus promoting antigen presentation [8–11]. In addition to the antigen-presenting process, CD40-activated signals are also involved in the priming of T cells [12], the cytotoxicity of T cells [13], the proliferation and differentiation of B cells, and immunoglobulin class switching and so on [14]. Although it participates in physiological processes, many studies have been published on its role in the pathology of disease. Activation of CD40 signaling is present in type 1 diabetes, multiple sclerosis (MS), inflammatory bowel disease (IBD), psoriasis, rheumatoid arthritis, and systemic lupus erythematosus (SLE) [15]. Disrupting the pathway has shown significant effects on the treatment of most of these diseases in mouse models (NOD, EAE, IBD, CIA and SLE mouse models) [16]. Several CD40L-CD40 interaction-blocking antibodies such as BG9588, IDEC-131 and ch5D12 have gone through or are undergoing clinical trials, and some have shown curative effects. Thus, the CD40 signaling pathway is considered to be a promising target for the clinical treatment of autoimmune diseases [17–19].

In this study, we aimed to disrupt the CD40L-CD40 interaction by expressing the extracellular domain of CD40. CD40-N, a 174 amino acids soluble form of the extracellular domain of CD40, was designed. A methylotrophic yeast called *Pichia pastoris* was used in this study as an efficient protein expression system to produce large amounts (g/L) of heterologous protein [20]. The induced protein was secreted into the culture supernatant and purified by size-exclusion chromatography and ion exchange chromatography. Finally, purified CD40-N was obtained with a purity of more than 90 %. The purified protein was able to block the CD40 activated signaling in vitro and to decrease the symptom of DSS-induced colitis in vivo. Thus, the purified CD40-N protein may be useful for further functional and structural studies.

## Methods

### Mice

Male C57BL/6 mice were purchased from Shanghai Laboratory Animal Center, Chinese Academy of Sciences (Shanghai, China). All mice were housed and maintained in SPF conditions. All animal experiments were performed in compliance with the Guide for the Care and Use of Laboratory Animals and approved by the Institutional Biomedical Research Ethics Committee of the Shanghai Institutes for Biological Sciences, Chinese Academy of Sciences.

### Strains, plasmids

The cell strain GS115 and the reconstructed plasmid pPIC9K were provided by the Key Laboratory of Molecular Medicine of Fudan University [21]. The *E. coli* strain DH5α was purchased from TIANGEN Biotech Co., Ltd (Beijing), and pcDNA3.3 was purchased from Invitrogen.

Yeast nitrogen base (with or without ammonium sulfate) was obtained from Sigma. Other reagents were of analytical purity. Sephadex G-50, and Q-Sepharose-FF were purchased from GE Healthcare.

### Construction of expression vector pPIC9K/CD40-N

CD40-N is the region from 61 bp to 579 bp in CD40 (NM_001250.4), encoding amino acids 21 to 193. A codon-optimized version of CD40-N was synthesized with *Xho*I and *Not*I sites at either end and cloned into the pUC57 plasmid by Sangon Biotech. The plasmid was digested with *Xho*I and *Not*I (Thermo Scientific) to release the CD40-N sequence. The sequence was then inserted into the yeast expression vector pPIC9K using the same restriction sites. The ligation product was transformed into *E. coli* DH5α competent cells. Successful recombinant colonies with pPIC9k/CD40-N were confirmed by restriction digest with *Xho*I and *Not*I and sequencing. Small-scale plasmid preparations, restriction digests, ligations and transformations were performed according to the manufacturer’s protocols.

### Transformation of *P. pastoris* to produce a CD40-N-expressing strain

The constructed plasmid pPIC9K/CD40-N was linearized with *Sal*I. The digested product was purified using an EasyPure PCR Purification Kit (Transgene Biotech) and used to transform the yeast host strain GS115. The transformation was carried out by electroporating *P. pastoris* as described in the *Pichia* expression manual (Invitrogen). Briefly, the GS115 cells were cultured in YPD medium until the OD_600_ reached 0.6–0.8. Then, the cells were pelleted by centrifugation at 3000 rpm for 5 min. Competent cells were generated by washing the cells twice with ice–cold water and followed by washing twice with ice-cold D-sorbitol buffer (1 M). Finally, the competent cells were resuspended in 1 mL of D-sorbitol buffer mixed with linearized plasmid in an electroporation cuvette on ice before electroporation (Micropulser™ Bio-Rad). Transformed cells were supplied with 1 mL ice-cold D-sorbitol immediately after electroporation and cultured at 30 °C for 1 h. The transformants were plated on MD plates (2 % glucose, 4 × 10^−5^ % biotin, and 1.34 % YNB) for 2–3 days.

Approximately 800 colonies on the MD plate were selected and screened for G418 (Amresco E859-5G) resistance. First, colonies were synchronized twice by culturing in 200 μL YPD medium in a 96-well plate for 24 h. Then, colonies were screened in media containing 1 mg/mL G418 for 24 h. Positive colonies (those that grew on the G418 plate) were cultured in a new plate with medium containing a higher concentration of G418 (2 mg/mL) for 24 h. This procedure was repeated until the strain could not grow on the plate. Strains that could grow at the highest concentration of G418 were stored at −80 °C for further experiments.

To induce the expression of CD40-N, each clone was streaked onto an YPD plate to obtain single colony. The single colony was then inoculated in 50 mL of BMGY in 250 mL flasks and cultured at 30 °C with 220 rpm shaking. When the OD_600_ reached 3–4, cells were harvested by centrifugation and briefly rinsed with water to remove trace glycerol. Rinsed cells were centrifuged and re-suspended in 50 mL BMMY. The cells were cultured in a new 250-mL flask at 30 °C with 220 rpm shaking and supplemented with methanol to a final concentration of 1 % every 24 h. 80 μL supernatant sample was collected every 24 h to examine the expression of CD40-N by SDS-PAGE and Western blotting.

### Large-scale production of CD40-N

Large-scale production of CD40-N was carried out using the clone that had the best yield in the small-scale experiments. A single colony was selected and grown in 5 mL of YPD medium at 30 °C with 220 rpm shaking overnight. The overnight culture was diluted (1:40) into 200 mL YPD medium and grown at 30 °C, 220 rpm shaking until an OD_600_ of 4.0 was reached. The culture was transferred into 3 L of medium in a bioreactor (Bioflow 3000 NBS) and grown in batch mode for 20 h. A sharp increase in dissolved oxygen (DO) occurred when the OD_600_ reached 70, suggesting that the glycerol was exhausted. Glycerol (50 %, v/v) was fed at a rate of 20 mL/(L · h) until the OD_600_ reached 110. The methanol-fed phase began once all of the glycerol was consumed. The methanol feed rate gradually increased from 0.8 to 4 mL/(L · h) in the first 6 h, allowing the culture to adapt to methanol consumption. After 6 h, the methanol feed rate was maintained at 4 mL/L^.^h for an additional 30 h.

The fermentation medium (1 L) contained 1.5 g sodium citrate•2H_2_0, 1.01 g CaSO_4_•2H_2_0, 18 g K_2_SO_4_, 7.32 g MgSO_4_, 4.13 g KOH, 27 mL 85 % H_3_PO_4_, 32 mL glycerin and 2 mL PTM1 solution. A PTM1 solution was added to the fermentation medium with 2 mL/L. The PTM1 (1 L) solution contained 6 g CuSO_4_•5H_2_O, 3 g MnSO_4_•H_2_O, 0.02 g H_3_BO_4_, 20 g ZnCl_2_, 0.8 g KI, 0.2 g NaMoO_4_•2H_2_O, 0.49 g CoCl_2_•6H_2_O, 65.06 g FeSO_4_•7H_2_O, 10 mL H_2_SO_4_, 0.5 g CaSO_4_•2H_2_O, 1.71 g MgSO_4_, and 0.2 g biotin, and the solution was sterilized with a 0.22 μm filter (Merck Millipore MPGL04001).

### Purification of the CD40-N protein

The supernatant from the fermentation was loaded into an ultra-filtration system (Merck Millipore, P2B005A05) to concentrate it to approximately 500 mL. Then, the sample was run through a Sephadex G-50 size-exclusion column that had been pre-equilibrated with at least 2 CV (column volumes) of buffer A (20 mM Tris-HCl, pH 7.4). Then, the fraction containing the protein was applied to a Q-Sepharose-FF (2 cm × 50 cm) column at 5 mL/min using an ÄKTA explorer 100. The column was washed with buffer A until the UV 280 (nm) was at the base level. Then, a linear gradient of buffer B (1.0 mol/L NaCl, 20 mM Tris-HCl pH 7.4) was used to elute the protein from the column. The protein concentration was measured by the BCA assay. The protein sample was lyophilized and stored at −80 °C.

### Coomassie blue staining and western blots

SDS-PAGE analysis was performed using 12 % gels according to the standard method. The entire gel was stained in Coomassie blue staining solution overnight before being placed in destaining buffer. For Western blotting, the proteins on the gel were transferred to a polyvinylidene difluoride membrane (Immobilon P, Millipore) using a wet electroblotting apparatus (Bio-Rad) at 100 V for 50 min in a solution of Tris-glycine (25 mM Tris, 192 mM glycine). The membrane was blocked by incubating with 5 % non-fat milk for 1 h at RT. Then, the membrane was immunoblotted with primary antibody at 4 °C overnight and with HRP-conjugated secondary antibodies for 1 h at RT. Detection of the bound antibody was performed using Super Signal West Pico Chemiluminescent Substrate (Pierce). The primary antibody against CD40-N (AF632) was purchased from R&D Systems. The anti-Flag antibody was purchased from Sigma (F3165). Mouse monoclonal antibodies against CD40 (G28-5 and 3A8) were purchased from ATCC. Anti–NF-κB2 (#4882) was purchased from Cell Signaling Technology (Danvers, MA). Anti–glyceraldehyde-3-phosphate dehydrogenase (GAPDH) monoclonal antibody was purchased from Kangchen (KC-5G4, Shanghai, China).

### Sugar content analysis of the recombinant protein

Purified CD40-N was treated with PNGase F (New England Biolabs P0704S) according to the manufacturer’s protocol, and the treated protein was analyzed by SDS-PAGE.

### ITC_200_ protein protein interaction assay

The cell is filled with G28-5 at 1 mg/mL, and the syringe is filled with CD40-N at 6 mg/mL. At special time intervals (150 s), a small volume (2 μL) of the CD40-N solution is injected into the cell triggering the binding reaction and producing the characteristic peak sequence in the recorded signal (Fig. 6a), during time of each drop was 4 s, and 19 drops were injected.

### Biological activity assay

BJAB cells were plated at a density of 10^6^ cells/mL and cultured in RPMI 1640 medium supplemented with 10 % FBS and 2-mercaptoethanol (Invitrogen) in 12-well plates. CD40-N was added at different concentrations of 0, 100, 300, 500 μg/mL at the same time with G28-5 (10 μg/mL). 1 h later, cells were harvested and qRT-PCR was performed to detect the RNA levels of Bcl-xL and TNF-alpha. CD40L was purchased from Peprotech (310–02).

### RNA extraction and Real-time polymerase chain reaction

Total RNA was isolated from cell lines using SuperfecTRITM, Total RNA Isolation Reagent (Shanghai Pufei Biotech Co., Ltd, 3101–100) according to the manufacturer’s protocol. To obtain cDNA, reverse transcript was performed using PrimeScript^TM^ RT reagent Kit with gDNA Eraser (TaKaRa, RR047A) according to the manufacturer’s instructions and 400 ng RNA was used as template. Quantitative real-time PCR (qRT-PCR) were performed using a 7500 Fast Realtime PCR System (Applied Biosystems, Carlsbad, CA), and all qRT-PCR reagents and consumables were purchased from Applied Biosystems and TaKaRa. For each reaction, reverse transcript product was diluted 10 times and 5 μL of the products was added to a 20 μL reaction system (TaKaRa, RR420A). Other reagents including predesigned and synthesized forward and reverse primer were added according to the manufacturer’s protocol and a two steps method was performed. Each sample was analyzed in triple replication. Relative quantification (RQ) was derived from the difference in cycle threshold (Ct) between target gene and actin (△Ct) using the equation RQ = 2^-△Ct^. The levels of mRNA were quantitatively assessed by SYBR Green-based quantitative PCR with gene specific primers. Actin was used as control. The primers were as follow:

- human *ACTIN* forward primer CTGGAACGGTGAAGGTGACA,
- human *ACTIN* reverse primer AAGGGACTTCCTGTAACAATGCA;
- human Bcl-xL forward primer CTGCTGCATTGTTCCCATAG,
- human Bcl-xL reverse primer GACGAGTTTGAACTGCGGTA;
- human TNF-alpha forward primer CAGAGGGAAGAGTTCCCCAG
- human TNF-alpha reverse primer CCTTGGTCTGGTAGGAGACG.

Error bars represent SD, and statistical significance calculated using two-tailed, unpaired *t* test.

### DSS-induced colitis

Male C57BL/6 mice were fed for 5 days with drinking water containing dextran sulfate sodium (DSS) (M.W. 36000–50000 Da; MP Biomedical, #160110) at a concentration of 2.75 % (w/v), and then allowed to recover by drinking regular water for another 2 days. The animals were weighted daily and monitored for signs of rectal bleeding.

## Results

### Construction, expression, and detection of CD40-N

The DNA fragment encoding the partial human CD40 gene (21–193 aa), CD40-N, underwent codon optimization according to yeast’s preference and was inserted between the *Xho*I and *Not*I sites of the expression vector pPIC9K. The correct sequence of the recombinant was confirmed by DNA sequencing.

**Fig. 1:**
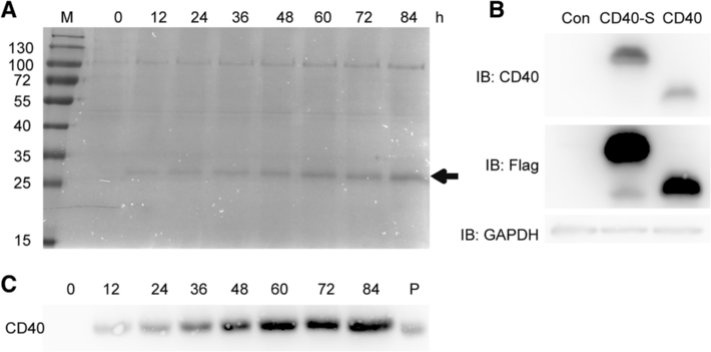
SDS–PAGE and Western blot analysis of recombinant CD40-N expressed in *P. pastoris*. **a** A 20-μL sample of supernatant was loaded onto a 12 % SDS–PAGE gel and stained with Coomassie brilliant blue G-250. All of the SDS-PAGE experiments were performed under the same conditions. Samples were collected at 12-h intervals for 84 h. The sizes of molecular weight markers (kDa) are shown (M), and recombinant CD40-N protein is indicated with an arrow. **b** Western blot analysis of human Flag-tagged CD40-N (not undergoing codon optimization) and CD40-N-S (with signal sequence and not undergoing codon optimization) expressed in HEK293T cells. pCDNA3.3-CD40-N and pCDNA3.3-CD40-N-S were introduced into HEK293T cells, and the cell lysates were harvested after 48 h. A 10-μl sample was loaded onto a 12 % SDS–PAGE gel. Anti-Flag, anti-CD40-N and anti-GAPDH antibodies were used to detect the proteins. **c** Western blot analysis of proteins at different times in *P. pastoris* (0, 12, 24, 36, 48, 60, 72, and 84 h). P indicates the positive control (CD40-N expressed in HEK293T cells)

Transformation of *P. pastoris* with pPIC9K yielded 4 transformants that were able to grow in the presence of a high concentration of geneticin, including three strains that grew in 4 mg/mL geneticin on YPD plates and one strain that grew in 1 mg/mL geneticin. After methanol induction, the supernatants were harvested and analyzed by Coomassie blue staining and Western blotting. Coomassie blue staining detected an increasing band at approximately 27 kDa that peaked at 96 h, suggesting that CD40-N may be secreted into the culture medium (the theoretical size of CD40-N is 19.3 kDa; Fig. 1a). Western blots were performed after CD40-specific antibody (AF632) was checked. A human CD40-N (not undergoing codon optimization, with or without signal sequence) gene fragment was cloned into pcDNA3.3 plasmid with a Flag tag at the C-terminus. The constructs were transferred to HEK293T cells and expressed for the Western blot assay. The CD40 antibody detected the same band with the Flag antibody, demonstrating the specificity of the antibody (Fig. 1b). The antibody was used to detect the culture medium, and Western blotting results showed that the potential CD40-N band in Coomassie blue staining was specifically recognized by the CD40-specific antibody, indicating that the protein expressed by *P. pastoris* was recombinant CD40-N (Fig. 1c). Comparing the band intensities of different clones by Coomassie blue staining and Western blot analysis showed that clone number 1 had the highest expression level of CD40-N.

### Fermentation and purification of CD40-N

**Fig. 2:**
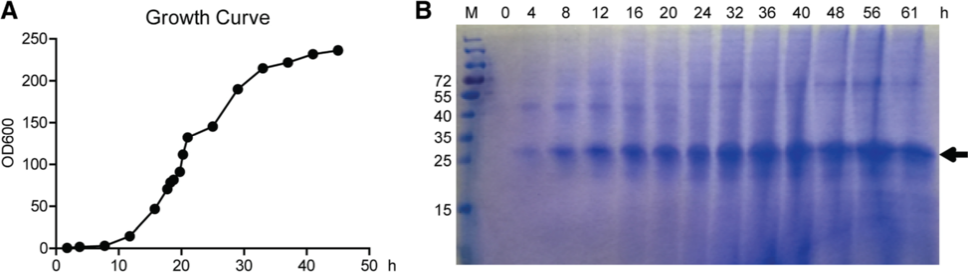
Fermentation of the CD40-N-expressing strain. **a** Growth curve of the *Pichia* culture. **b** Time course of CD40-N expression in the bioreactor. Fermentation supernatants were collected at the indicated times after methanol induction, and recombinant CD40-N protein is indicated with an arrow

**Fig. 3:**
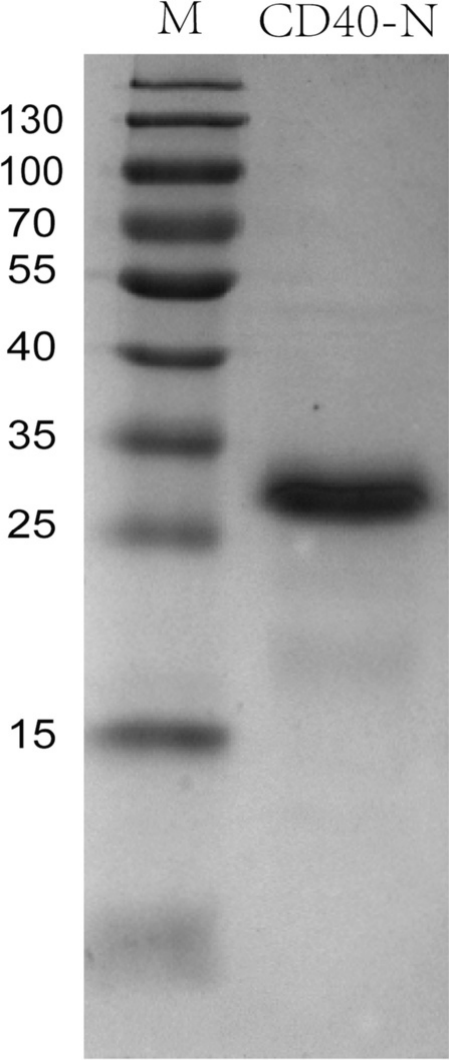
Purification of CD40-N. 1: Marker; 2: sample after purification

*P. pastoris* clone NO.1 was grown in a 5 l stirred bioreactor, as described in the Methods section, growth curve of the yeast was shown in Fig. 2a the amounts of expression of CD40-N was peaked at 36 h and then be kept stable (Fig. 2b). After 36 h of fermentation, the supernatant, approximately 3 l, was concentrated to approximately 500 mL after ultrafiltration using a 5 kDa membrane. Then, the 500 mL sample was applied to a flow-through Sephadex G50 size-exclusion column to be desalted and purified from smaller proteins. After that, ion-exchange chromatography was used to obtain high-purity protein. A total of 120 mg protein with a purity of more than 90 % was collected at the washing step using approximately 0.03 M NaCl in buffer Tris-HCl (pH 7.4) [22] (Fig. 3).

### Sugar content of the recombinant protein

**Fig. 4:**
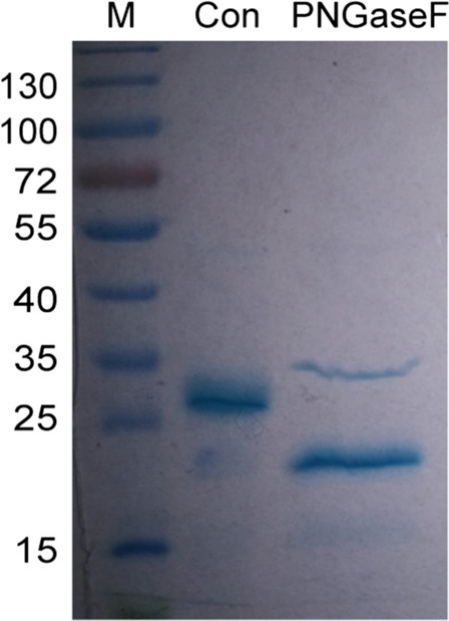
Glycosylation analysis of CD40-N. M: sizes of molecular weight markers (kDa); PNGase F, treatment of the protein with PNGase F; Con, treatment of the protein without PNGaseF

To study the glycosylation of the purified CD40-N, the purified protein was treated with the deglycosylating enzyme PNGase F, which is derived from *Flavobacterium meningosepticum* and can remove N-linked carbohydrates. As shown in Fig. 4, Coomassie blue staining detected a single band at a molecular weight of 27 kDa before deglycosylation and a band of approximately 19.3 kDa after treatment. This indicates that the purified CD40-N was N-glycosylated. The band at approximately 35 kDa was PNGase F [23].

### ITC assay proved the interaction of CD40-N and G28-5

**Fig. 5:**
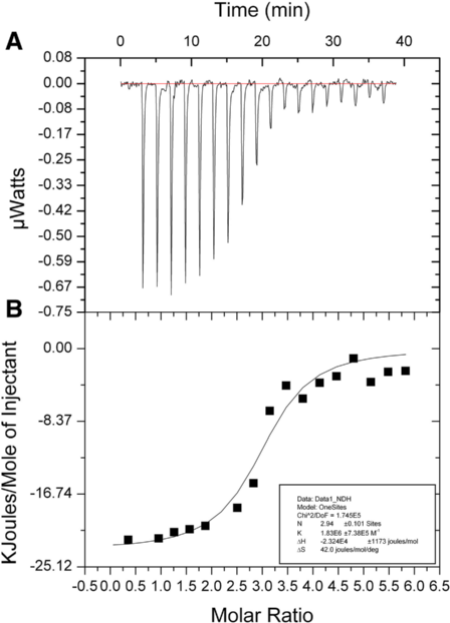
ITC assay reveals the interaction of CD40-N with CD40-specific antibody, G28-5. **a** The heat effects of CD40-N and G28-5; **b** After integration of area under each peak (and normalization per mol of injected protein), the thermodynamic parameters was calculated using nonlinear regression analysis

To study whether G28-5, a CD40 agonist antibody, can bind CD40-N directly. We use ITC_200_ system to analysis the interaction [24]. The process was performed according to the method. After saturating the macromolecule, the residue heat effects are due to mechanical and dilution effects (Fig. 5a). After the integration of the area of each peak, the individual heats are plotted against the molar ratio from which through nonlinear regression (Fig. 5b), the thermodynamic parameters was calculated, K_D_ =0.546 μmol, and △H =−23.24KJ/mol.

### CD40-N can disrupt CD40-activated signaling

**Fig. 6:**
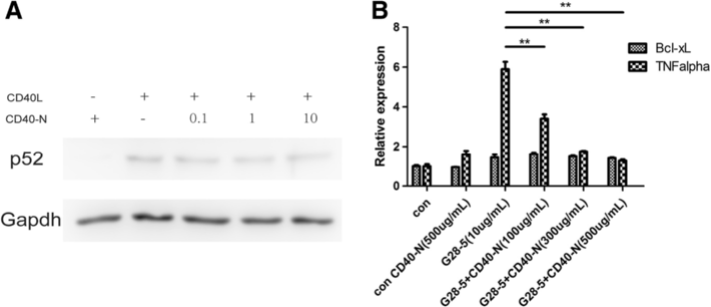
CD40-N disrupts the interaction of CD40 and CD40L or CD40-specific agonist antibody *in vitro.* **a** BJAB cells were stimulated with CD40L at 24 h in the presence of titrated purified CD40-N protein (0, 0.1, 1, 10 μg/mL). Western blot was performed to examine p100 procession into p52. **b** BJAB cells were stimulated with G28-5 (10 μg/mL) for 1 h with or without CD40-N. Real-time PCR was performed to examine the gene expression

The recognition of CD40 by CD40 ligand or CD40-spcific agonist antibody results in NFκB p100 processing into NFκB p52, activates alternative NFκB signaling and induces downstream target gene expression. To determine whether the purified protein CD40-N can disrupt the interaction of CD40 with CD40L or CD40-specific agonist antibody, we treated BJAB cells with CD40L in the presence of titrated CD40N. Western Blot showed that CD40-N reduced the p100 processing into p52 induced by CD40L in BJAB cells in a dose-dependent manner (Fig. 6a). G28-5 can activate CD40 signaling and further induce the expression of downstream target genes such as TNF-alpha. We observed that CD40-N significantly reduced TNF-alpha mRNA level induced by CD40-specific agonist antibody, G28-5, in a dose dependent manner (Fig. 6b), while the RNA levels of Bcl-xL was not induced by G28-5 as negative control. These data demonstrate that the purified protein CD40-N could disrupt the interaction of CD40 with CD40L or CD40-specific agonist antibody in vitro.

### Functional CD40-N can relieve the symptoms of DSS-induced colitis

**Fig. 7:**
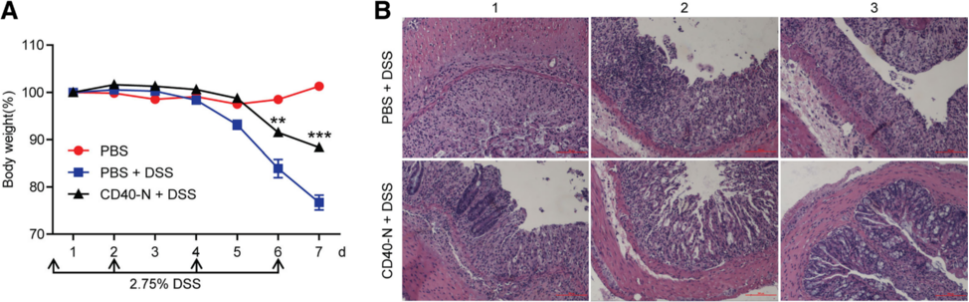
CD40-N administration reduces the symptom of DSS-induced colitis. **a** Wild-type C57bl/c mice were injected on day 0, 2, 4, and 6 with either PBS or 200 μg of CD40-N and were given 2.75 % DSS. Body weight loss was recorded every day. The data represent the mean ± SEM (DSS with CD40-N: *n* = 6; DSS with PBS: *n* = 5; without DSS: *n* = 3). Student’s *t*-test was performed for statistical analysis. **p < 0.01, ***p < 0.001. **b** H&E staining of colon sections of mice at 7 day. The three pictures in the upper panel come from 3 DSS-treated mice with PBS treatment. The pictures in the lower panel show 3 DSS-treated mice with CD40-N treatment (magnification: 200×)

CD40 signaling has been shown to play an important role in inflammations such as colitis [25–28]. To determine whether CD40-N functions in vivo, we employed a DSS-induced colitis mouse model to test it. At 7-day post-treatment, the mean body weight of the PBS treated mice was reduced to 76.8 % of the starting weight; while the CD40-N treatment could significantly recover the loss of body weight (88.4 %, Fig. 7a). H&E staining indicated that damage to the architecture of the colon in CD40-N treatment mice was reduced compared to that of the control mice, and crypt atrophy and cilia damage were both less prevalent (Fig. 7b). These data reveal that CD40-N reduced inflammation in vivo to alleviate the symptoms of DSS-induced colitis in mice.

## Discussion

Targeting CD40L-CD40 interaction could be useful in clinical applications for curing autoimmune diseases, providing treatment following transplantation and treating tumors [15]. One strategy to disrupt this interaction is to use an anti-CD40L monoclonal antibody: this approach has been shown to be effective in mouse models of RA, SLE, MS, IBD, T1 diabetes, and inflammatory heart disease [29]. The humanized CD40L monoclonal antibody BG9588 (hu5c8) has shown therapeutic effects on SLE patients in clinical trials [17, 30]. Another humanized monoclonal antibody, IDEC-131, was tested in a phase II clinical study in ITP patients [31]. However, they were not approved for clinical use because of thrombotic complications when BG9588 was used in some SLE patients and IDEC-131 was used in treating Crohn’s disease [27]. A third humanized anti-CD40L antibody, ABI793, targeted a different epitope and was found to have the same thrombotic complications, suggesting that these complications are a common effect of anti-CD40L antibodies regardless of epitope specificity. Recently, researchers have found that the interaction of the Fc fragment of the anti-CD40L antibody with the Fc receptor CD32 in platelets may cause platelet cross-linking and lead to clotting [32]. This is consistent with the fact that thrombus formation was not observed in mouse models because mouse platelets do not express a homolog of CD32. Reconstruction of the Fc fragment of the antibody had been shown to eliminate the complications while maintaining the therapeutic effects of the anti-CD40L antibody in SLE and MS mouse models [33, 34]. The transformed isotype of high-affinity fragment Fab’ and F(ab)’2 has also been studied. All of these methods provide new insights into the effects of disrupting CD40L-CD40 interaction using a CD40L antibody.

Another strategy to disrupt this interaction is to target CD40. Some CD40 antibodies have been tested. HCD122, an antibody that can disrupt CD40L-CD40 interaction but cannot activate CD40 signaling, has been used in a clinical trial to treat CD40^+^ multiple myeloma because of its ADCC function [35]. Another antibody, ch5D12, has shown some curative effect in a phase II clinical study for the treatment of Crohn’s disease [19].

Targeting the CD40L-CD40 interaction is an important method of immunotherapy for cancer treatment. Dacetuzumab (or SGN-40, an anti-CD40 antibody) has been used in clinical trials for treating CLL, MM and NHL [36, 37].

Given the importance of the pathway activated by CD40 in research and its clinical applications, we constructed a CD40-N expression system in *P. pastoris. P. pastoris* was chosen because of its high production yield, expression stability, ability to secrete proteins, moderate post-translational modifications, and simple economical culture conditions. Additionally, this expression system has been used to prepare many recombinant proteins for research and clinical applications [20, 38].

Recombinant CD40-N was purified from the culture medium by a combination of Sephadex G-50 size-exclusion and Q FF–Sepharose ion exchange chromatography. The purity of the final recombinant CD40-N exceeded 90 %. ITC assay verified the interaction of CD40-N with CD40-specific antibody. The purified CD40-N showed biologically activity based on its ability to reduce CD40L-activated non-canonical NF-κB signaling pathway and inhibit TNF-alpha expression induced by CD40-specific agonist antibody in a dose-dependent manner in vitro. Importantly, CD40-N protein could significantly decrease the inflammation in DSS-induced colitis mouse model. These data reveal that we have established a reliable method for the expression and purification of CD40-N, which is functional in vitro and in vivo in interrupting the interaction of CD40 and CD40L.

Because CD40 is a glycoprotein, glycosylation may play a critical role in its structure and function. We showed that our recombinant CD40-N was glycosylated with N-linked sugars, which was responsible for the increased molecular weight observed by SDS-PAGE (from 19.3 to 27 kDa). The predicted potential N-linked glycosylation sites of CD40-N are Asn153 and Asn180, and the exact N-linked sugars sites remain to be elucidated.

## Conclusions

This work has successfully generated CD40-N recombinant protein in *Pichia pastoris* that can disrupt the CD40L-CD40 interaction. It may serve as a foundation for further scientific and clinical research. The protein fragment we have acquired has potential to be used in research or even treating inflammation diseases such as colitis.

## Abbreviations

ADCC: Antibody-dependent cell-mediated cytotoxicity
BMGY: Buffered glycerol-complex medium
BMMY: Buffered methanol-complex medium
CIA: Collagen-induced arthritis
CLL: Chronic lymphocytic leukaemia
EAE: Experimental autoimmune encephalomyelitis
HRP: Horseradish peroxidase
IBD: Inflammatory bowel disease
ITP: Idiopathic thrombocytopenic purpura
MM: Multiple myeloma
MS: Multiple sclerosis
NHL: Non hodgkin lymphoma
OD: Optical density
PTM1: Trace salts medium
qRT-PCR: Quantitative real-time polymerase chain reaction
RT: Room time
SDS-PAGE: SDS-polyacrylamide gelelectrophoresis
SLE: Systemic lupus erythematosus
SPF: Specific-pathogen-free
TCR-MHC: T cell receptor-major histocompatibility complex
TNF: Tumor necrosis factor
YPD: Yeast extract peptone dextrose medium

## References

[1] Grewal IS, Flavell RA (1998). CD40 and CD154 in cell-mediated immunity. Annu Rev Immunol.

[2] Alderson MR, Armitage RJ, Tough TW, Strockbine L, Fanslow WC, Spriggs MK (1993). CD40 expression by human monocytes: regulation by cytokines and activation of monocytes by the ligand for CD40. J Exp Med.

[3] Karmann K, Hughes CC, Schechner J, Fanslow WC, Pober JS (1995). CD40 on human endothelial cells: inducibility by cytokines and functional regulation of adhesion molecule expression. Proc Natl Acad Sci U S A.

[4] Hollenbaugh D, Mischel-Petty N, Edwards CP, Simon JC, Denfeld RW, Kiener PA (1995). Expression of functional CD40 by vascular endothelial cells. J Exp Med.

[5] Banchereau† CvKaJ: CD40-CD40 ligand. J Leukocyte Bio.l 2000, 67. 10.1002/jlb.67.1.2 10647992

[6] Hostager BS, Bishop GA (2013). CD40-Mediated Activation of the NF-kappaB2 Pathway. Front Immunol.

[7] Vallabhapurapu S, Karin M (2009). Regulation and function of NF-kappaB transcription factors in the immune system. Annu Rev Immunol.

[8] Noelle RJ, Ledbetter JA, Aruffo A (1992). CD40 and its ligand, an essential ligand-receptor pair for thymus-dependent B-cell activation. Immunol Today.

[9] Foy TM, Shepherd DM, Durie FH, Aruffo A, Ledbetter JA, Noelle RJ (1993). *In vivo* CD40-gp39 interactions are essential for thymus-dependent humoral immunity. II. Prolonged suppression of the humoral immune response by an antibody to the ligand for CD40, gp39. J Exp Med.

[10] Van den Eertwegh AJ, Noelle RJ, Roy M, Shepherd DM, Aruffo A, Ledbetter JA (1993). *In vivo* CD40-gp39 interactions are essential for thymus-dependent humoral immunity. I. *In vivo* expression of CD40 ligand, cytokines, and antibody production delineates sites of cognate T-B cell interactions. J Exp Med.

[11] Clark EA, Ledbetter JA (1994). How B-Cells And T-Cells Talk To Each Other. Nature.

[12] Grewal IS, Xu J, Flavell RA (1995). Impairment of antigen-specific T-cell priming in mice lacking CD40 ligand. Nature.

[13] Bourgeois C, Rocha B, Tanchot C (2002). A role for CD40 expression on CD8+ T cells in the generation of CD8+ T cell memory. Science.

[14] Banchereau J, Bazan F, Blanchard D, Briere F, Galizzi JP, van Kooten C (1994). The CD40 antigen and its ligand. Annu Rev Immunol.

[15] Peters AL, Stunz LL, Bishop GA (2009). CD40 and autoimmunity: the dark side of a great activator. Semin Immunol.

[16] Grewal IS (2009). Overview of TNF superfamily: a chest full of potential therapeutic targets. Adv Exp Med Biol.

[17] Boumpas DT, Furie R, Manzi S, Illei GG, Wallace DJ, Balow JE (2003). A short course of BG9588 (anti-CD40 ligand antibody) improves serologic activity and decreases hematuria in patients with proliferative lupus glomerulonephritis. Arthritis Rheum.

[18] Davis JC, Totoritis MC, Rosenberg J, Sklenar TA, Wofsy D (2001). Phase I clinical trial of a monoclonal antibody against CD40-ligand (IDEC-131) in patients with systemic lupus erythematosus. J Rheumatol.

[19] Kasran A, Boon L, Wortel CH, Hogezand RA, Schreiber S, Goldin E (2005). Safety and tolerability of antagonist anti-human CD40 Mab ch5D12 in patients with moderate to severe Crohn's disease. Aliment Pharmacol Ther.

[20] Macauley-Patrick S, Fazenda ML, McNeil B, Harvey LM (2005). Heterologous protein production using the Pichia pastoris expression system. Yeast.

[21] Mo W, Zhang YL, Chen HS, Wang LS, Song HY (2009). A novel hirudin derivative characterized with anti-platelet aggregations and thrombin inhibition. J Thromb Thrombolysis.

[22] Huang Y, Zhang Y, Wu Y, Wang J, Liu X, Dai L (2012). Expression, purification, and mass spectrometric analysis of 15 N, 13C-labeled RGD-hirudin, expressed in Pichia pastoris, for NMR studies. PLoS One.

[23] Li H, Li N, Gao X, Kong X, Li S, Xu A (2011). High level expression of active recombinant human interleukin-3 in Pichia pastoris. Protein Expr Purif.

[24] Velazquez-Campoy A, Leavitt SA, Freire E (2015). Characterization of protein-protein interactions by isothermal titration calorimetry. Methods Mol Biol.

[25] Danese S, Scaldaferri F, Vetrano S, Stefanelli T, Graziani C, Repici A (2007). Critical role of the CD40 CD40-ligand pathway in regulating mucosal inflammation-driven angiogenesis in inflammatory bowel disease. Gut.

[26] Uhlig HH, McKenzie BS, Hue S, Thompson C, Joyce-Shaikh B, Stepankova R (2006). Differential activity of IL-12 and IL-23 in mucosal and systemic innate immune pathology. Immunity.

[27] Danese S (2004). The CD40/CD40L costimulatory pathway in inflammatory bowel disease. Gut.

[28] Visekruna A, Linnerz T, Martinic V, Vachharajani N, Hartmann S, Harb H (2014). Transcription factor c-Rel plays a crucial role in driving anti-CD40-mediated innate colitis. Mucosal Immunol.

[29] Law CL, Grewal IS (2009). Therapeutic interventions targeting CD40L (CD154) and CD40: the opportunities and challenges. Adv Exp Med Biol.

[30] Huang WQ, Sinha J, Newman J, Reddy B, Budhai L, Furie R (2002). The effect of anti-CD40 ligand antibody on B cells in human systemic lupus erythematosus. Arthritis Rheum.

[31] Kuwana M, Nomura S, Fujimura K, Nagasawa T, Muto Y, Kurata Y (2004). Effect of a single injection of humanized anti-CD154 monoclonal antibody on the platelet-specific autoimmune response in patients with immune thrombocytopenic purpura. Blood.

[32] Koyama I, Kawai T, Andrews D, Boskovic S, Nadazdin O, Wee SL (2004). Thrombophilia associated with anti-CD154 monoclonal antibody treatment and its prophylaxis in nonhuman primates. Transplantation.

[33] Ferrant JL, Benjamin CD, Cutler AH, Kalled SL, Hsu YM, Garber EA (2004). The contribution of Fc effector mechanisms in the efficacy of anti-CD154 immunotherapy depends on the nature of the immune challenge. Int Immunol.

[34] Nagelkerken L, Haspels I, van Rijs W, Blauw B, Ferrant JL, Hess DM (2004). FcR interactions do not play a major role in inhibition of experimental autoimmune encephalomyelitis by anti-CD154 monoclonal antibodies. J Immunol.

[35] Tai YT, Li X, Tong X, Santos D, Otsuki T, Catley L (2005). Human anti-CD40 antagonist antibody triggers significant antitumor activity against human multiple myeloma. Cancer Res.

[36] Forero-Torres A, Furman RR, Rosenblatt JD, Younes A, Harrop K, Drachman JG (2006). A humanized antibody against CD40 (SGN-40) is well tolerated and active in non-Hodgkin's lymphoma (NHL): Results of a phase I study. J Clin Oncol.

[37] Furman RR, Forero-Torres A, Shustov A, Drachman JG (2010). A phase I study of dacetuzumab (SGN-40, a humanized anti-CD40 monoclonal antibody) in patients with chronic lymphocytic leukemia. Leuk Lymph.

[38] Thompson CA (2010). FDA approves kallikrein inhibitor to treat hereditary angioedema. Am J Health Syst Pharm.

